# Toll-like receptor 3 as an immunotherapeutic target for *KRAS* mutated colorectal cancer

**DOI:** 10.18632/oncotarget.16812

**Published:** 2017-04-04

**Authors:** Radhashree Maitra, Titto Augustine, Yitzchak Dayan, Carol Chandy, Matthew Coffey, Sanjay Goel

**Affiliations:** ^1^ Albert Einstein College of Medicine and Montefiore Medical Center, Bronx, NY 10461, USA; ^2^ Oncolytics Biotech Inc., Calgary, AB T2N 1×7, Canada

**Keywords:** Toll-like receptor, reovirus, colorectal cancer, innate immunity, immunotarget

## Abstract

New therapeutic interventions are essential for improved management of patients with metastatic colorectal cancer (mCRC). This is especially critical for those patients whose tumors harbor a mutation in the *KRAS* oncogene (40-45% of all patients). This patient cohort is excluded from receiving anti-EGFR monoclonal antibodies that have added a significant therapeutic benefit for *KRAS* wild type CRC patients. Reovirus, a double stranded (ds) RNA virus is in clinical development for patients with chemotherapy refractory *KRAS* mutated tumors. Toll Like Receptor (TLR) 3, a member of the toll like receptor family of the host innate immune system is the pattern recognition motif for dsRNA pathogens. Using TLR3 expressing commercial HEK-Blue™-hTLR3 cells we confirm that TLR3 is the host pattern recognition motif responsible for the detection of reovirus. Further, our investigation with *KRAS* mutated HCT116 cell line showed that effective expression of host TLR3 dampens the infection potential of reovirus by mounting a robust innate immune response. Down regulation of TLR3 expression with siRNA improves the anticancer activity of reovirus. *In vivo* experiments using human CRC cells derived xenografts in athymic mice further demonstrate the beneficial effects of TLR3 knock down by improving tumor response rates to reovirus. Strategies to mitigate the TLR3 response pathway can be utilized as a tool towards improved reovirus efficacy to specifically target the dissemination of *KRAS* mutated CRC.

## INTRODUCTION

Aberrant cell signaling cascades are the hallmark of cancer [1, 2]. One such oncogenic mutation is that of *KRAS* which is found in 40-45% of all human metastatic colorectal cancers (mCRC) [3]. This gene has been marked to be the first genetic biomarker by US Food and Drug Administration [4]. A *KRAS* mutation in codons 12, 13, 61, and 146, are now valid markers to exclude the use of the anti-EGFR monoclonal antibodies in patients with mCRC. The lack of alternate therapeutic strategies for *KRAS* mutated CRC patients thus makes this an area of imminent clinical need and urgent exploration.

Reovirus, an oncolytic dsRNA virus is in current clinical development with over 30 active/completed clinical trials to its credit. Reovirus has shown safety and efficacy in clinical trials, and till date is best developed in combination with chemotherapy. Constitutive *KRAS* signaling is a prevalent phenomenon that occurs in diverse tumor types and is associated with transformation, proliferation, and reduced sensitivity to conventional chemotherapy [5]. The scientific rationale for the development of reovirus as an anticancer agent stems from the fact that it preferentially replicates and induces lysis of cells with a mutated *KRAS* pathway [6]. This fact has been further exploited by therapeutic administration of reovirus in patients with *KRAS* mutated CRC [7]. However, none of the multiple clinical trials that are underway and completed have shown complete response to the virotherapy. One approach to improve the efficacy of reoviral therapy is to use it in conjunction with approved chemotherapy or radiotherapy. Other plausible approach is to harness cellular mechanism that would offer a better possibility of virus delivery or a favorable environment for virus mediated cellular dissemination.

The innate immune system possesses a skillful organization comprising of pattern recognition receptors (PRRs) that sense invasion of microbial pathogens by toll like receptors (TLRs). Foreign nucleic acids, the signature of invading viruses, are sensed at the intercellular level [8]. In particular the recognition of dsRNA is achieved by endosomal TLR, namely TLR3 [9]. During viral infection, cells initiate antiviral responses to contain replication and inhibit virus spread. The protective mechanism signaled by TLR3 is furthered to stimulation and activation of transcription factors, namely interferon regulatory factor 3 (IRF-3) and NF-κB, resulting in secretion of the antiviral cytokine, interferons [10]. It has also been reported that primary intestinal epithelial cells of normal mucosa constitutively expressed TLR3 and TLR5, while TLR2 and TLR4 were only barely detectable [11]. The exact mechanism and specificity of recognition of reovirus by the toll like receptors is yet to be reported.

It has been elucidated that reovirus engage cells by binding to cell-surface carbohydrates along with the immunoglobulin super family member, junction adhesion molecule-A (JAM-A). Following attachment, reovirus internalization is promoted by β1 integrins, most likely via clathrin-dependent endocytosis [11]. Although the mechanism of reovirus entry to the host cells has been critically elucidated, its recognition and interactions with endosomal TLRs and the trigger mechanism of innate immune system remains unknown.

In the current study we establish that reovirus is indeed recognized by TLR3 recognition pathway. Down regulation of TLR3 in the CRC cell line HCT116 which constitutively harbors *KRAS* mutation improves the anti-cancer activity of the reovirus. We furthered our investigation into a xenograft murine model and have observed a similar improvement in the anti-cancer activity of reovirus in TLR3 down regulated tumors. This phenomenon can effectively be harnessed in enhancing the oncolytic properties of reovirus with improved therapeutic outcome.

## RESULTS

### TLR3 is confirmed to be the host pattern recognition motif for dsRNA containing reovirus

**Figure 1:**
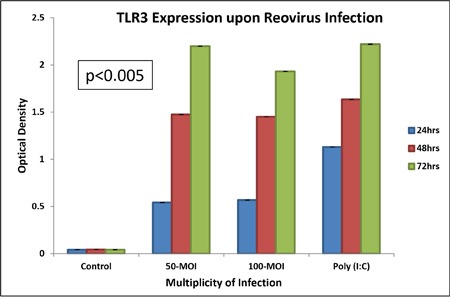
We confirm that TLR3 is involved in reovirus infection using HEK-Blue™ hTLR3 cells transfected with pUNO-TLR3 plasmid engineered with an inducible SEAP reporter gene At 50 MOI of reovirus infectivity, TLR3 is induced steadily with increase in time as is measured by colorimetric assay of activation of alkaline phosphatase reporter gene under the control of NF-Kβ promoter. The “y” axis represents the optical density of alkaline phosphatase reporter gene expression.

TLR3 expressing commercial HEK-Blue™-hTLR3 cells were treated with reovirus at 50 multiplicity of infection (MOI) or poly (I:C), the positive control for TLR3 to confirm that TLR 3 is the host pattern recognition motif of the cellular machinery that is responsible for the detection of dsRNA reovirus (Figure 1). The expression was significantly (p<0.005) increased at 24 hours, further doubled at 48 hours and continued to show an upward trend up to 72 hours. Increasing the concentration of infective particles to 100 MOI did not improve the expression pattern and it appears that MOI 50 is a saturating concentration of infection for this particular cell line which was obtained by stable transfection of HEK293 cells with a pUNO-TLR3 plasmid that expresses the TLR3 gene. The reporter assay was based on SEAP (embryonic alkaline phosphatase) secreting expression plasmid under the control of NF-kB promoter.

### The gene expression of TLR3 and TRIF is enhanced upon reovirus infection

**Figure 2:**
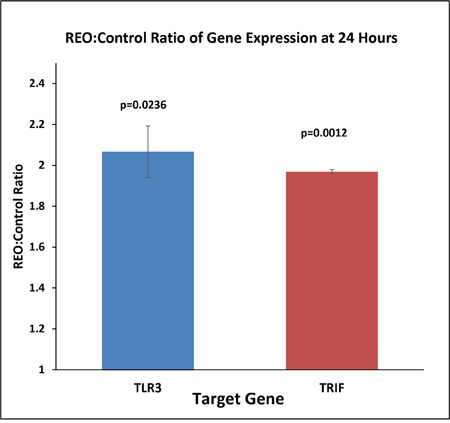
The cDNA was prepared from 500ng of total RNA extracted from reovirus treated (5MOI) and control HCT116 cells at 24 hours Real time (RT) PCR was performed with TLR3 and GAPDH primers and an average of 2.4 fold increase in expression of both TLR3 and TRIF was observed at 24 hrs post infection when the expression was normalized to GAPDH expression. The fold change was significant with SEM of 0.218 (p=0.023).

To confirm the pattern of gene expression of TLR3 and TRIF (TIR Domain-Containing Adapter Protein Inducing IFN-β) an intermediate adapter protein in TLR3 signaling pathway, we performed a quantitative real time PCR with cDNA synthesized from total RNA obtained from control and reovirus (5 MOI) treated HCT116 colorectal cancer cell line at 24 hours. The gene expression pattern showed a two fold increase for both the genes at 24 hours (Figure 2, p=0.0236 for TLR3, and p=0.0012 for TRIF). This confirmed that the TLR3 is not only the pattern recognition motif for the dsRNA of reovirus but the virus recognition is actually translated to downstream triggering intermediate adaptor protein TRIF.

### Down regulation of TLR3 in *KRAS* mutated CRC enhances reovirus mediated cell killing

**Figure 3:**
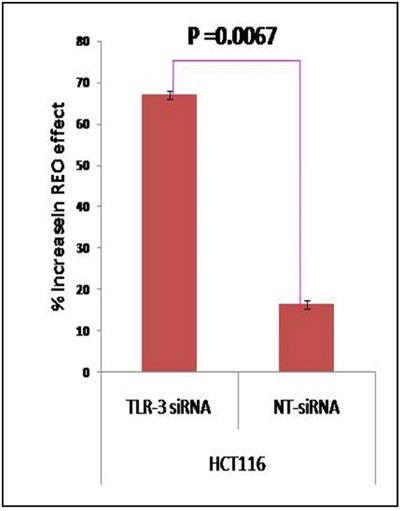
CRC cell line HCT116 was transiently transfected with TLR3 specific siRNA and NT siRNA At 6 hours post transfection, the cells were infected with reovirus (5 MOI) and MTT assay was done at 48 hours. The graph represents the mean percent increase in reovirus' anti-cancer activity upon TLR3 siRNA down-regulation as compared to non-targeted siRNA determined by MTT Assay. The siRNA enhanced reovirus cell killing of HCT116 is improved significantly (p= 0.0067).

TLR3 has been reported to be constitutively expressed by colonocytes [12]. To further confirm that TLR3 is the reovirus recognizing receptor for HCT116 colon cancer cell line, we next down regulated TLR3 in the cell line using small interfering (si) RNA followed by reovirus treatment at dose of 5 MOI for 48 hours (Supplementary Figure 1). The MTT assay clearly indicates that HCT116 cells with silenced TLR3 (67.15%) experienced a significantly greater inhibition of cell proliferation as compared to cells transfected with non-targeted (NT) siRNA (16.48%, p=0.0067; Figure 3). Thus TLR3 which is constitutively expressed by colon epithelium is also a mediator of virus recognition in CRC cell line HCT116.

### Release of Type I and II interferons are significantly reduced in TLR3 down regulated HCT116 cells upon virus challenge

**Figure 4:**
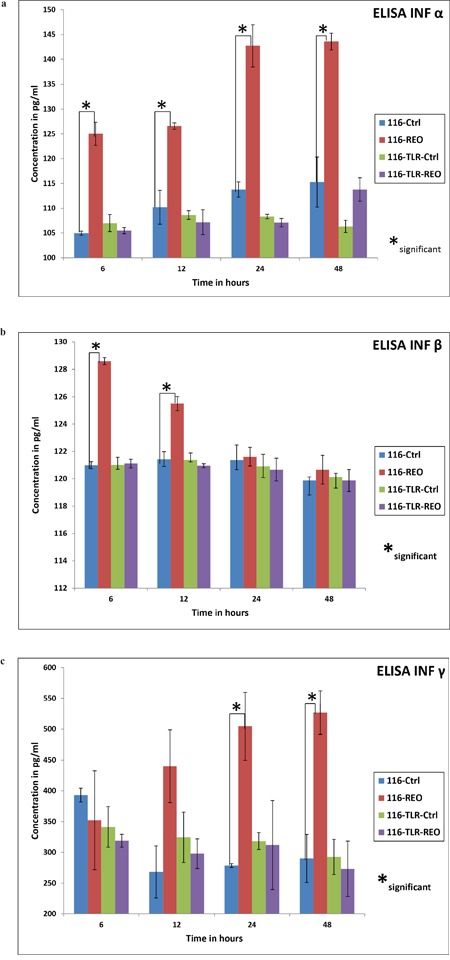
The ELISA for IFN-α,-β and -γ are shown in order in three graphs **(a)**, **(b)** & **(c)**. TLR-3 down regulated cells showed no increase in release of the soluble cytokines upon reovirus infection at a dose of 5 MOI. On the contrary, HCT116 control cells did show a rise in release of the three classes of interferons upon reovirus infection at 5 MOI. In case of IFN-α there was a significant increase (p≤ 0.05) of cytokine productions at all 4 time points namely 6, 12, 24 and 48 hours as represented by *. In case of IFN-β the significance (p≤ 0.05) was observed in control cells treated and untreated at 6 and 12 hours where as the IFN-γ showed a significant difference in the same group at 24 and 48 hours.

To assess the ultimate downstream effect of TLR3 down regulation, an ELISA assay of INF α, INF β and INF γ was performed. Essentially, HCT116 control cells and those which were transfected with TLR3 specific siRNA or NT siRNA were treated with reovirus at a dose of 5 MOI. The supernatant was collected at 6, 12, 24, and 48 post virus treatment. ELISA was performed to quantify the secretion of INF α, INF β and INF γ. When treated with reovirus, HCT116 cells showed a significant release of INF α at 6 hours post infection which steadily increased up to 24 hours and continued to maintain the concentration at 48 hours (Figure 4a, 4b, 4c). INF β, on the other hand had peaked at 6 hours, followed by a gradual drop, and returned to basal level by 24 hours (Figure 4b). INF γ expression was first noted 12 hours post infection with persistent increase up to 48 hours (Figure 4c). On the contrary the TLR3 suppressed HCT116 cells when treated similarly with reovirus did not secrete any of the interferons (INF-α, INF-β and INF-γ) beyond the basal level. The release of all 3 interferons into the supernatant by unmodified HCT116 cells was significantly higher than with TLR3 silencing. The release of interferons by NT siRNA treated HCT116 cells were similar to unmodified HCT116 cells (data not shown). These three assays distinctly reveal that downstream activation of the respective interferons was completely compromised post reovirus infection in TLR3 down regulated HCT116 cells.

### RNA sequence analysis confirms unaltered expression of TLR3 between *KRAS* mutant and wild type colorectal cancer cell lines

**Figure 5:**
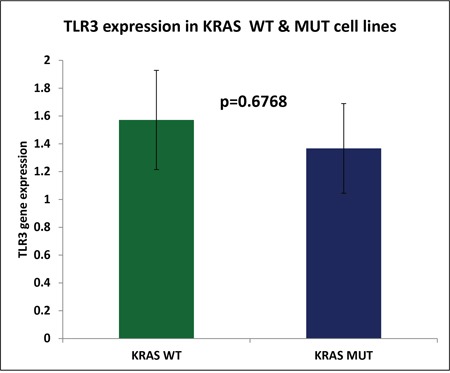
Represents the average TLR3 expression of *KRAS* wild type cell lines (n=23) and *KRAS* mutant CRC cell lines (n=33) as measured by RNA sequencing analysis The difference in TLR3 expression was determined to be insignificant (p=0.6768).

We have previously reported that reovirus is selectively more potent as an oncolytic agent in *KRAS* mutated CRC cells as compared to the *KRAS* wild type CRC cells [13]. To confirm that the mutational status of *KRAS* does not contribute to TLR3 down regulation paving the path for better virus mediated oncolysis we quantified the TLR3 expression in 56 CRC cell line (23 *KRAS* wild type CRC cells and 33 *KRAS* mutant) as obtained from transcriptome sequencing [14]. Indeed, TLR3 expression was not significantly different between *KRAS* mutant and WT cell lines (p=0.66; Figure 5).

### TLR3 down regulated HCT116 xenografted tumors showed pronounced tumor regression upon reovirus administration

Subcutaneous xenograft tumors were generated on the right flank of 8 weeks old athymic mice. 24 athymic mice were divided into 3 groups of 8 mice each; each group received 5 million cells of either HCT116, or TLR3 silenced (TLR3 specific shRNA transfected) HCT116, or plasmid A transfected HCT116 cells. Within each group, 4 animals were left untreated and 4 received a daily dose of 1×10^7^ TCID_50_ (median Tissue Culture Infectivity Dose) of reovirus intratumorally. Each experiment was repeated three times.

**Figure 6:**
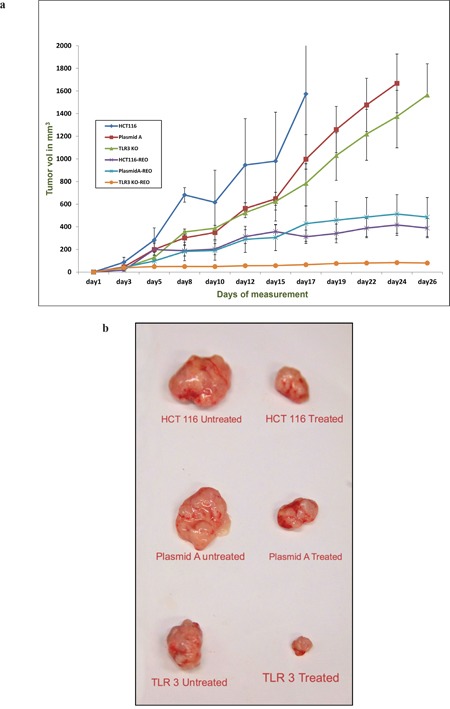
**(a)** The graph represents the mean tumor volume of each of the six group of animal namely HCT116, HCT116-plasmid A, HCT116 TLR3 knockout (KO) and reovirus treated versions of the aforementioned cell line derived xenografts (n=4). The tumors were measured thrice a week and a total of 12 measurements were taken. Day 1 represents the initiation of reovirus treatment. All animals were alive till the end point of experiment. **(b)** The photograph of representative excised tumors from each of the six groups showing the comparative tumor size growth inhibition upon reovirus treatment at the endpoint of the study.

In mice injected with untreated HCT116 cells, the mean [+/- standard error of the mean (SEM)] tumor weight of the mice treated with reovirus was 620 (+/- 22) mg, and was significantly lower than the tumor growth in the untreated mice, 2560 (+/- 45) mg (p = 0.000074; 75.8% reduction; Figure 6). In mice injected with plasmid A treated HCT116 cells, the mean (+/- SEM) tumor weight of the mice treated with reovirus was 600 (+/- 79) mg, and was significantly lower than the tumor growth in the untreated mice, 1673 (+/- 73) mg (p = 0.0052; 58% reduction; Figure 6). In mice injected with TLR3 down regulated HCT116 cells, the mean (+/- SEM) tumor weight of the mice treated with reovirus was 115 (+/- 8) mg, and was significantly lower than the tumor growth in the untreated mice, 1698 (+/- 84) mg (p = 0.000081; 93.2% reduction; Figure 6). Clearly, reovirus is an effective anti cancer agent, and demonstrated a significant reduction tumor weights in all 3 models.

We next assessed the degree of response in the 3 different model systems. The effect of reovirus on the plasmid A transfected cells generated tumors very closely resembled the HCT116 generated tumors (p = 0.93). However, the effect of reovirus was significantly higher in the TLR3 silenced HCT 116 cells generated tumors than plasmid A transfected cells generated tumors (p = 0.000042), and untreated HCT 116 cells generated tumors (p = 0.0000085) (Figure 6b and Supplementary Table 1). This clearly demonstrates *in vivo*, the effect of TLR3 knockdown on reovirus activity.

### Histological analysis of the tumor specimen indicates greater extent of necrosis in TLR3 down regulated xenografts upon reovirus administration

**Figure 7:**
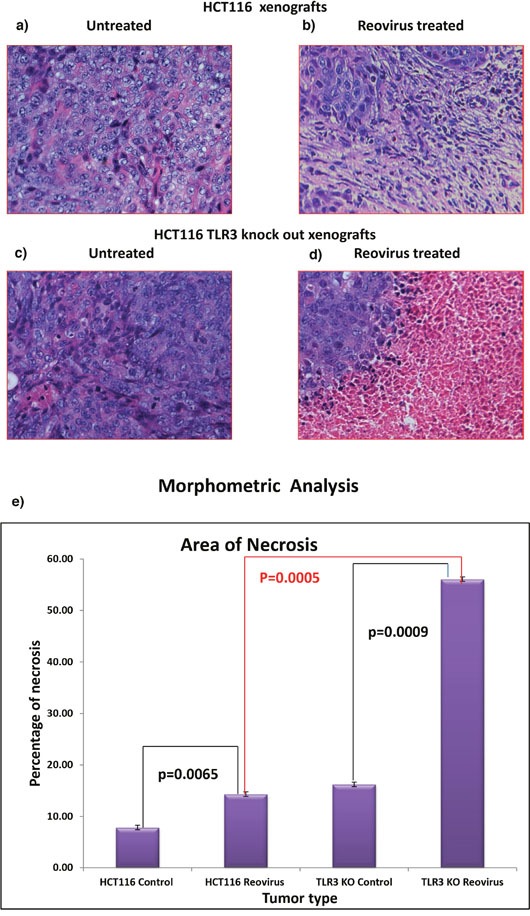
**(a)** & **(b)** H&E stained tumor photograph at 20X magnification of tumor generated by HCT116 upon reovirus treatment. No area of necrosis is observed. **(c)** & **(d)** H&E stained tumor photograph at 20X magnification of tumor generated by TLR3 silenced HCT116 cells followed by upon reovirus treatment. Distinct pink area of necrotic region is observed with islands of tumor cells demonstrating that the virus caused better dissemination of the tumor cells. **(e)** graphical representation of morphometric analysis to quantify the area of necrosis in reovirus treated xenografts. We documented that there is significant increase in necrosis both in reovirus treated HCT116 xenografts as well as HCT116-TLR3 KO xenografts when compared to untreated (p=0.0065 & p=0.0009) group. Furthermore, the extent of necrosis was significantly greater in HCT116-TLR3 KO when compared to HCT116 xenograft treated with reovirus.

The cellular morphology was similar in the untreated tumors generated by HCT116 cells (Figure 7a and 7c) and those generated by TLR3 knocked down HCT116 cells. However, in post reovirus treated tumors the TLR3 silenced tumors depicted a better response with large regions of necrosis (Figure 7b and 7d) as observed by hematoxylin and eosin staining. While the reovirus treated HCT116 cells showed uniform distribution of scar tissues, the TLR3 silenced tumors showed mostly necrotic tissue with pronounced eosin (pink) staining indicating formation and accumulation of acidophilic dead tissue. These data clearly indicate that the mode of tumor cell killing by reovirus is altered upon TLR3 knockdown, with a greater degree of tissue necrosis and reduced vascular density. To quantify the extent of necrosis we performed a morphometric analysis (Figure 7e) which confirmed a significant increase (p=0.0005) in necrotic area in reovirus treated HCT116-TLR3 knocked down xenografts when compared to reovirus treated HCT116 xenografts.

### Increased expression of reoviral sigma1 capsid protein, and RNA dependent RNA polymerase lambda3 protein in reovirus treated TLR3 KO cells

**Figure 8:**
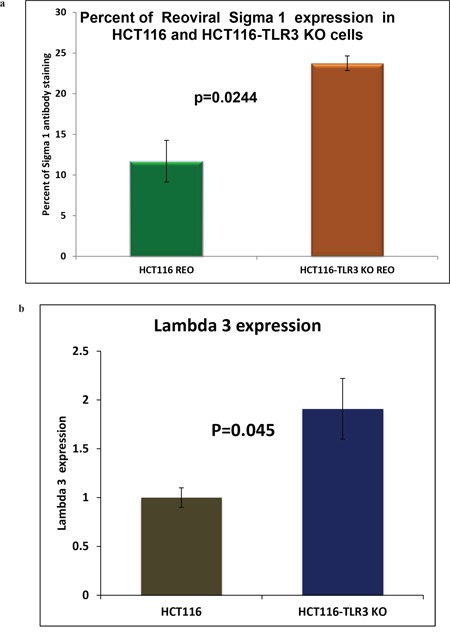
**(a)** Morphometric analysis of IHC stained HCT116 cells and HCT116-TLR3 KO cells 24 hours post reovirus treatment with antibody raised against reovirus sigma 1 capsid protein. There is a 1.85 -fold increase in expression of the sigma1 protein in TLR3 KO cells (p=0.0244). **(b)** Graphical representation of the expression of RNA dependent RNA polymerase lambda 3 in HCT116 cells and HCT116-TLR3 KO cells 24 hours post reovirus treatment as measured by real time PCR. A 1.91-fold increase in expression was observed in the TLR3 KO cells. (p=0.045).

To further confirm that there is an increased reovirus replication in the TLR3 knocked down condition, we demonstrate increased viral protein expression. We performed two different experiments. We first checked the expression of viral capsid protein Sigma 1 by IHC followed by morphometric analysis for quantification (Figure 8a). We have observed a 1.85-fold increase in the expression of sigma 1 protein in reovirus treated HCT116 TLR3 KO cells at 24 hours with a significant p value of 0.0244. We next performed real time PCR to quantify the expression of RNA-dependent RNA polymerase Lambda 3 in HCT116 and HCT116 TLR3 KO cells at 24 hours’ post reovirus infection. We observed a 1.91 fold increase in expression of Lambda 3 in HCT116-TLR3 KO cells. (p=0.045).

## DISCUSSION

Treatment of patients with colorectal cancer whose tumors bear a *KRAS* mutation remains a therapeutic challenge to this day. This cohort represents approximately 40-45% of all CRC patients. It has been universally accepted that the presence of a *KRAS* mutation is a negative predictive marker of the two USFDA approved anti-EGFR monoclonal antibodies, namely cetuximab and panitumumab [15]. Thus, development of alternate treatment options for patients with *KRAS* mutated CRC is an absolute necessity.

Reovirus, a ubiquitous dsRNA virus has gained importance in the recent years because of its oncolytic properties specifically in *KRAS* mutated environment. The use of reovirus in combinatorial studies with approved chemotherapy and radiation have shown promising results both in animal models as well as in clinical trials [7, 16]. One approach to improve the efficacy of reovirus therapy is to use it in conjunction with approved chemotherapy or radiotherapy, as is being tested in clinical trials. The other plausible approach is to harness the cellular mechanism that could offer a better possibility of virus delivery and a favorable environment for virus mediated cellular dissemination. As such, a molecularly detailed understanding of the host-virus interaction is essential. While both pre-clinical and clinical data have demonstrated that the virus is active and definitely so in *KRAS* mutant cancers [7, 13], its mechanism of activity remains elusive. *KRAS* mutation is fairly common, particularly in pancreas, colon, thyroid and lung cancers [3, 6, 17]. The major focus of this investigation was based upon our preliminary finding that *KRAS* mutated CRC cell presented a better candidacy for reovirus mediated oncolysis [13] and this is clinically relevant as the treatment options are currently limited for this cohort of CRC patients [18]. We propose to develop an alternate treatment modality wherein attempts are made to improve the efficacy of the virus by enhancing the virus delivery. One mode of improving the delivery can be achieved by the down regulation of the virus recognizing host receptor that is responsible for triggering the initial antiviral immune response.

Till date the plausible contribution of TLR and orchestrated modulation of innate immune system remains largely unexplored. TLRs are mediators of inflammation in the gut and potentially important modulators of colon and rectal cancer risk. The intestinal epithelial cells of normal mucosa constitutively expressed TLR3 [11, 12, 19]. TLR3 is essentially a pattern recognition receptor for dsRNA and an important member of the innate immune system. It is proposed that upon recognizing and binding to the dsRNA it should initiate a cascade of signals which would trigger the production of interferon and other cytokines and finally activate the NF-κβ working its way towards stimulating the adaptive immune system [12]. To date, little is known about reovirus’ recognition by host cellular machinery. It is clearly evident from our study that down regulation of TLR3 supports active viral propagation with significantly reduced host immune trigger as indicated by lower cytokine response. This is a desirable situation within the *KRAS* mutated tumor for a more efficient virus mediated cell killing. Our study explores and harnesses this phenomenon in *ex vivo* and preclinical study platform.

In the current study we first established that TLR3 is activated on exposure to reovirus. Using HEK293 cells that are genetically engineered with TLR3 gene under the control of NF-kB promoter along with a SEAP reporter assay system we established that reovirus is recognized by TLR3. After confirming that TLR3 is the receptor for recognizing reovirus we proceeded to confirm that TLR3 is constitutively expressed in CRC cell line by transcriptome analysis. This analysis gave us the confidence that TLR3 is constitutively expressed in the colon epithelium. Furthermore, we clearly verified that the expression of TLR3 by colon epithelia is independent of the *KRAS* mutation status. It is clearly evident from our study that down regulation of TLR3 supports active viral propagation with significantly reduced host immune trigger. We did a quantitative MTT assay with untreated and reovirus treated HCT116 cells in which TLR3 expression was down regulated by siRNA and established that virus induces a higher extent of growth inhibition in TLR3 silenced CRC cells. This is a desirable situation within the *KRAS* mutated tumor for a more efficient virus-mediated cell growth inhibition.

The expression of TLR3 and TRIF, an important intermediate of TLR3 signaling pathway were assayed by RT-PCR. Expression of both proteins was doubled at 24 hours when compared to untreated cells. The expression of two different viral proteins namely reovirus capsid protein sigma 1 and RNA dependent RNA polymerase lambda3 when quantified in HCT116 and HCT116-TLR3 KO cells clearly documented near doubling in expression. Furthermore we established that reovirus recognition by TLR3 stimulates a signaling cascade. The type-I interferon (IFN-α/β) response is critical to immunity against viruses and can be triggered in many cell types by cytosolic detection of viral infection [20]. We performed ELISA for type I (IFN-α/β) and type II (IFN-γ) and documented loss of secretion of these interferons upon virus challenge inTLR3 down regulated cells. We thus established that TLR3 signaling pathway is the primary host immune response in CRC cell lines when challenged with reovirus.

To further establish the role of TLR3 in virus recognition and the fact that silencing of TLR3 expression can be an alternate treatment modality we furthered our investigation to *in vivo* pre-clinical animal models. Nude mice were injected with tumor cells that were either unmodified or TLR3 silenced. Upon reovirus treatment of the tumors we observed a greater degree of tumor growth inhibition in TLR3 down-regulated tumor as compared to tumors with unmodified TLR3. The histological analysis revealed that reovirus treatment of TLR3 knocked out tumor has a different morphology than those with unmodified HCT116 cells. Although the untreated tumors were morphologically similar, the reovirus treated ones were different. The HCT116 cells generated tumors showed more scar tissues with some fine vascularization upon reovirus administration while the cells with TLR3 knockdown showed large areas of necrosis probably indicating a greater destruction of tumor tissues by necrosis thus making the tumors better contained. This finding essentially validates our hypothesis that knocking down the expression of the reovirus recognizing receptor can be an alternate method to improve the reovirus efficacy. Investigating the consequences of down regulation of toll receptors and subsequent alterations of efficacy of virotherapy is presently one of the major thrust areas demanding immediate scientific attention [24, 25].

Over the last decade, emphasis has shifted from empirical treatment of patients to a biomarker-led, precision approach. Discoveries in carcinogenesis, particularly within the field of molecular pathology, have shaped the personalized medicine paradigm [26]. In this context TLR3 can serve as an excellent therapeutic target for patients with *KRAS* mutated CRC as reovirus is more effective in *KRAS* mutated environment and further down regulation of TLR3 would augment to its efficacy thus being more specifically effective in the *KRAS* mutated patient cohort.

There are several TLR agonists available with few being FDA approved as adjuvant for immune stimulations in cancer patients [27–29]. A commonly recognized one is BCG that is widely used in the management of superficial urinary bladder cancer. BCG is an attenuated strain of Mycobacterium bovis that was primarily developed as a vaccine against tuberculosis. On the contrary, not too many TLR antagonists are available and none has been tested for toxicity or safety. Antagonists for TLR 3, 4 and 9 are being developed for research purposes but are yet far from being ready for therapeutic testing. Although there is no well established antagonist of TLR3 certain small molecules are being tested to check the efficacy of these molecules in effective TLR3 down regulation [30]. The other strategy of down regulation of TLRs can be achieved by developing and administrating neutralizing antibodies directed against specific TLRs [31]. This area requires much investigation in conjunction with molecular strategies towards improving the therapeutic capabilities of the oncolytic virus.

Our study attempts to understand the recognition and interaction of reovirus with its intracellular recognition receptor TLR3. Our findings clearly show that the dampening of TLR3 expression makes *KRAS* mutated cancer cells a better therapeutic target for oncolytic reovirus. This study warrants the necessity to delineate at the molecular level the mechanism adopted by TLR3 in reovirus infection and propagation with special emphasis on *KRAS* mutated CRC condition as a progress towards search of a better therapy of *KRAS* mutated CRC.

## MATERIALS AND METHODS

### Cell lines, culture conditions and virus

Human TLR3 expressing HEK293 cells were purchased from Invivogen (#hkb-htlr3) and HCT116 which harbors *KRAS* mutation was purchased from ATCC. HEK293 cells were cultured in DMEM, 4.5 g/l glucose, 2-4 mM L-glutamine, 10% (v/v) fetal bovine serum (FBS), 50 U/ml penicillin, 50 μg/ml streptomycin, 100 μg/ml Normocin™. HCT116 and Hke3 cells were cultured in MEM (Gibco BRL), supplemented with 10% FBS, 2mM L-Glutamine and 1% penicillin streptomycin and grown at 37°C at 5% CO_2_ concentration.

Reovirus type 3 dearing strain (trade name Reolysin^®^) was provided by Oncolytics Biotech Inc. (Calgary, Canada) at a TCID_50_ of 4.5 × 10^10^ particles per ml concentration. Virus particles were stored in the dark at -80°C for long term storage and at 4°C for up to 4 weeks. Appropriate dilutions were performed in growth media immediately prior to initiation of infection. Cells were infected for 6 hours followed by a change of media and infected cells were grown for a further 24-72 hours at 37°C. Polyinosinic-polycytidylic acid (poly(I:C), a synthetic analog of double-stranded RNA (dsRNA), a molecular pattern associated with viral infection. Poly(I:C) is recognized by TLR3 inducing the activation of NF-kB and the production of cytokines. Poly(I:C) (Invivogen # rtp-htlr3) was used at a concentration of 2 uM.

### Reovirus doses and administration

Reovirus was provided at a concentration of 4.5 × 10^10^ TCID_50_ stock solutions. It was serially diluted and a dose of 100 MOI (multiplicity of infection) was used for human TLR3 expressing HEK293 cells where as a MOI of 5 was used for HCT116 cells and its derivative with HCT116 cells with TLR3 silenced. In animal studies, reovirus was administered intratumorally (IT) at a daily dose of 10 million TCID_50_ (tissue culture infectious dose). The stock was diluted immediately before use and the leftover particles were appropriately discarded after treating with 10% bleach solution.

### Reporter assay for TLR3 activation

HEK-Blue™-hTLR3 cells are commercially developed by co-transfection of the human TLR3 gene and an inducible SEAP (secreted embryonic alkaline phosphatase) reporter gene into HEK293 cells. Essentially the SEAP gene is placed under the control of the IFN-β minimal promoter fused to five NF-κB and AP-1-binding sites. Stimulation with a TLR3 ligand activates NF-κB and AP-1 which induce the production of SEAP. Levels of SEAP were determined with HEK-Blue™ Detection system (hkb-htlr3, hb-sel) the cell culture medium that allowed for real-time detection of SEAP and provided a fast and convenient method to monitor SEAP expression. SEAP is secreted into cell culture supernatant and therefore did not require the preparation of cell lysates and was quantified using a microtiter plate reader in a spectrophotometer at 477 nm.

### Quantitative real time PCR to measure gene expression of TLR3 and TRIF and Reovirus Lambda 3

Real time quantitative PCR was performed with TLR3 primers (Invivogen # rtp-htlr3), TRIF and GAPDH primers (Supplementary Table 2) to quantify the expression of respective genes upon reovirus treatment for 24 hours. Essentially 500ng of RNA (Qiagen RNAeasy mini kit# 74104) was isolated from HCT116 cells harvested from each culture condition (control and reovirus-treated) at 24 hours and was used to make cDNA with Iscript cDNA synthesizing kit (Bio-Rad #1708890). Real time PCR was performed in 96 well qPCR plate using Affymetrix VeriQuest Fast SYBR Green qPCR Master Mix (2X) (Affymetrix part #75690) as fluorescent dye for detection. Bio-Rad CFX96 RT-PCR machine was used to amplify the cDNA. The Data was exported, derived and analyzed. TLR3 and TRIF expression were normalized to GAPDH expression, and then a ratio between the control and reovirus-treated samples was calculated.

Identical procedure was followed to measure the expression of reovius RNA polymerase Lambda 3 transcript using primers detailed in Supplementary Table 2 and normalized to the expression of GAPDH.

### Silencing of TLR3

TLR3 siRNA along with control NT siRNA as well as TLR3 shRNA plasmid (h) and control plasmid A were purchased from Santa Cruz Biotech (sc-36685-SH) and HCT116 and Hke3 cells were transfected with either TLR3 siRNA/NT siRNA or TLR3 shRNA plasmid along with control plasmid A shRNA as per the manufacturers protocol. Transfected cells were incubated 6 hours at 37°C in a CO_2_ incubator. Following incubation, 1 ml of normal growth medium containing 2 times the normal serum and antibiotics concentration (2x normal growth medium) was added and incubated for an additional 18-24 hours under conditions normally used to culture the HCT116 cells. The selection of stably transfected cells, was achieved with puromycin antibiotic (sc-108071) selection at 10 μg/ml. Approximately in every 2-3 days, the culture media was aspirated and replaced with freshly prepared selective media. TLR3 siRNA was essentially used for *in vitro* MTT assays, and stably transfected TLR3 shRNA/Plasmid A shRNA cells were utilized for cytokine ELISA and animal xenograft experiments.

### MTT assay for cell proliferation

For determination of reovirus sensitivity, 10,000 cells (HCT116/HCT116-TLR3 silenced along with Hke3/ Hke3-TLR3 silenced) per well were seeded in 96-well plates and treated with reovirus at 0, 5 MOI for 24, 48 and 72 hours. For each cell line, one plate was harvested at the time of viral infection for determination of t = 0 absorbance values. Viable cells were determined post treatment using the 3-(4,5-dimethylthiazol-2-yl)-2,5-diphenyltetrazolium bromide (MTT) (Sigma M2128) assay by measurement of absorbance at 570 nm [32]. The relative rate of cell growth for each cell line was factored into the analysis by subtracting the absorbance at time 0hrs from both the control and treatment groups. All experiments were repeated at least three times.

### ELISA

The cytokine assays were performed with the HCT116 control (untransfected) along with TLR3 and plasmid A shRNA transfected cells under both reovirus treated and untreated conditions. The supernatant were collected at 6, 12, 24 and 48 hrs. Human IFN-α serum reagent kit (PBL assay sciences # 41110-1 Verikine) was used to determine the level of IFN-α, and Human IFN-β (PBL assay sciences #41410-1A Verikine) was used to determine the levels of IFN-β. Levels of IFN-γ was determined using ENZO (cat No ADI-900-136) IFN-γ (Human) ELISA Kit. Each assay was repeated twice and was performed strictly following the manufacturers protocol. Essentially to the pre-wet wells the standards and the samples supernatants were added and incubated for 1 hour. The wells are then washed three times with the wash buffer supplied with the reagent kit and the appropriate detection antibody was added and incubation was continued for another 2 hours. The wells were again washed and the appropriate secondary antibody was added followed by incubation for another 30 mins. This was followed by addition of substrate and 15 mins incubation finally followed by addition of stop solution and reading at ELISA plate reader (Applied Biosystems) at 450 nm.

### RNA sequence analysis

High-throughput mRNA sequencing (RNA-seq) offers the ability to quantify genes, analyze transcriptomes and measure transcript expression in a single assay as described elsewhere [33]. Essentially cDNA was synthesized and put to high throughput transcriptome sequencing for the 56 CRC cell lines analyzed for this study. The 56 cells lines were separated on the basis of *KRAS* status. The constitutive expression of TLR3 was computed in the two groups and the mean and standard error of mean was determined. The detailed methodology of the high throughput sequencing and the cutoff and threshold are detailed in the following reference [14].

### Animal studies

Athymic nude mice (Harlan Laboratories # nu69) females, 8-9 wk old were divided into six groups (4 animals in each group) sedated with isoflurane gas and 5 million of either HCT116 or HCT116 –TLR3 silenced (shRNA: Santa Cruz # sc-36685-SH) or HCT116 transfected with Plasmid A (nonspecific shRNA Santa Cruz# sc-108060) cells mixed with matrigel (1:1) were injected using a 19.5 gauge sterile needle into the right flank of the animals. The tumor growth was monitored daily and when reached 100 mm^3^ in volume was given a treatment of reovirus at a daily dose on 10 million TCID_50_ administered intra-tumoral (IT). When the tumor size reached 2000 mm^3^ the animals were euthanized by carbon dioxide inhalation to effect. The animals were monitored for ulceration, bleeding or infection of the skin and tumor, and for decreased mobility or moribund status. The tumor length and width was measured thrice a week with digital calipers and the tumor volume was calculated using the formula Volume= A times B^2^ where A is the width and B is the length of the tumor. The experiment was repeated thrice. All experiments were conducted in accordance with the Institute for Animal Care and Use Committee of Albert Einstein College of Medicine.

### Hematoxylin and eosin staining of xenograft tumor

At the end point, animals were euthanized utilizing CO_2_. Tumors were excised and fixed in 10% neutral buffered formalin in PBS (pH 7.4) and placed at 4°C. After 24 h, samples were placed in 10% EDTA in PBS (pH 7.4) for 48–72 h at 4°C until decalcified. The tumors were then processed for paraffin embedding, sectioned to 5 μm, and stained with H&E. The slides were visualized and photographed at 20X magnifications on the Zeiss Axio Observer CLEM (Correlative Light and Electron Microscopy) at the AECOM Analytical Imaging facility.

### Immunohistochemistry

Reovirus treated (24 hours) HCT116 and HCT116-TLR KO cells were suspended in histo-gel and paraffin embedded. 10 um sections were stained with anti-goat antibody raised against reovirus sigma 1 protein (gift from Oncolytics Inc, Calgary Canada). Sections were incubated for 1 hour at 1:500 dilution of antibody and color was developed by 3,3′-Diaminobenzidine (DAB) standard immunohistochemical techniques. The stained slides were then subjected to morphometric analysis.

### Morphometry

Slides were scanned on the PerkinElmer P250 slide scanner (SIG #1S10OD019961-01) using a 20X objective. Brown stain analysis was completed on the whole piece of tissue on every slide with PerkinElmer's QuantCenter, using the DensitoQuant module. In this module, brown stain pixels were distinguished from the rest of the tissue by color thresh-holding. The analysis of pink versus purple areas of tissue was completed in imageJ, using the color threshold module.

### Western blot analysis

Reovirus treated and untreated cultured cells were collected, washed in PBS, and lysed in 150 mM Tris-HCl (pH 8), 150 mM NaCl, 5 mM EDTA and 1% Nonidet P-40 supplemented with a mixture of protease inhibitors (Roche, Basel, Switzerland). 400 micrograms total protein was pre-cleared and used to immune precipitate TLR3 protein using protein Agarose beads and TLR3 antibody (Antibody online # ABIN201783) for 24 hours at 4°C. The Agarose beads were washed thrice, eluted by boiling with Lameli buffer (Biorad 4x Laemmli Buffer #1610747) and run on a 12% SDS-PAGE. After blotting, membranes were probed with anti-TLR3 primary antibody (Antibody online # ABIN201783). Secondary antibodies were HRP-labeled, and detection was performed using the Clarity Chemiluminescent substrate (Bio-Rad # 1705061). Western blots were performed using standard procedures. Membranes were blocked with 5% milk in TBS containing 0.1% Tween 20, and incubated with antibodies specific for TLR3. Immunoreactive bands were visualized by chemiluminescence (# 1705061 Bio-Rad) clarity ECL western blotting detection kit.

## SUPPLEMENTARY MATERIALS FIGURE AND TABLES


