# Supplementary material for: Toll-like receptor 3 as an immunotherapeutic target for KRAS mutated colorectal cancer
Source: Oncotarget. 2017 Apr 4;8(21):35138–53. doi: 10.18632/oncotarget.16812 (PMC5471041; doi:10.18632/oncotarget.16812)
Supplement: Supplementary file 1 [file oncotarget-08-35138-s001.pdf]

Toll-like receptor 3 as an immunotherapeutic target for *KRAS* mutated colorectal cancer

SUPPLEMENTARY FIGURE AND TABLES

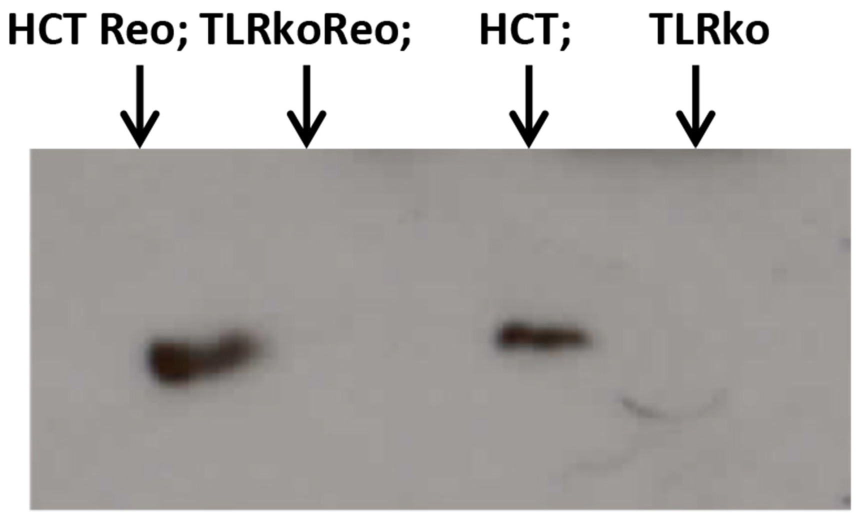

Supplementary Figure 1: The down regulation of TLR 3 was confirmed in both reovirus treated and untreated HCT116 and HKE3 cells by western blot analysis. 500 ugm protein was immunoprecipitated with TLR3 antibody.

Supplementary Table 1: Tabular representation of the mean tumor weight in grams of rodents in each study group (n=4)

	Mean tumor wt (in gm)	% reduction in tumor wt	T test TLR3 KO vs respective
**HCT116 untreated**	**2.5633**	**75.81**	**p = 0.0000085**
**HCT 116 treated**	**0.62**		
**Plasmid A untreated**	**1.6**	**57.97**	**p = 0.000042**
**Plasmid A treated**	**0.6725**		
**TLR3 KO untreated**	**1.6975**	**93.23**	
**TLR3 KO treated**	**0.115**		

The percent growth inhibition between the untreated and reovirus treated mice within each group and the significance as determined by T TEST.

Supplementary Table 2: List of Primers used in the study for real time PCR

hTRIF Forward	5’-ACGCCATAGACCACTCAGCTTTCA-3’
hTRIF Reverse	5’-AGGTTGCTCATCATGGCTTGGTTC-3’
hGAPDH Forward	5’TTCGACAGTCAGCCGCATCTTCTT-3’
hGAPDH Reverse	5’-GCCCAATACGACCAAATCCGTTGA-3’
Reo LAMBDA3 Forward	5’TTTTGCAACAAACTGCGGGT 3’
Reo LAMBDA3 Reverse	5’ ACATGCCATAGCGTCACGAA3’
hTLR3 Forward	# rtp-htlr3 Forward Invivogen
hTLR3 Reverse	# rtp-htlr3 Reverse Invivogen
